# A Clinical and Pathological Analysis of Seven Cases of Salivary Gland Ductal Carcinoma

**DOI:** 10.7759/cureus.105809

**Published:** 2026-03-25

**Authors:** Zhou Yan, Ziqing Tang, Shiyuan Li, Aijun Guo

**Affiliations:** 1 Stomatology, Northern Jiangsu People's Hospital, Yangzhou, CHN; 2 Stomatology, Clinical Medical College, Yangzhou University/Northern Jiangsu People’s Hospital, Yangzhou, CHN

**Keywords:** clinical pathology, immunophenotype, malignant salivary gland tumor, parotid gland, pathological analysis, pathological characteristics, pathological morphology, salivary gland ductal carcinoma

## Abstract

Background and objective

Salivary duct carcinoma (SDC) is a rare but highly aggressive malignancy. Due to its low incidence, most published data are derived from small retrospective series or case reports, and the optimal diagnostic work-up, prognostic stratification, and treatment algorithms remain poorly defined. This study aimed to investigate the clinical characteristics, pathological features, and treatment strategies for SDC by reviewing the relevant literature.

Methods

A retrospective analysis was conducted on the clinical and pathological data, histological morphology, immunophenotypic characteristics, and treatment outcomes of seven patients with SDC. Additionally, the relevant literature regarding SDC was also reviewed.

Results

Among the patients, six were male, and one was female, with a mean age at onset of 58 ± 13.84 years. Most cases occurred in the parotid gland (4/7), followed by the submandibular gland (2/7) and the upper lip mucosa (1/7). The tumors exhibited histological features similar to those of breast cancer, with atypical cells arranged in cribriform, nest-like, and linear patterns. The cells had large nuclei with prominent nucleoli, abundant eosinophilic cytoplasm, and an infiltrative growth pattern. Immunohistochemistry showed positive staining for CKpan, CK8/18, CEA, EMA, AR, 34βE12, and GATA3. The Ki67 proliferation index was high, whereas P63, P40, calponin, S100, SMA, and ER were negative. Some cases showed weak positivity for PR and strong positivity for HER2.

Conclusions

SDC is a rare but highly aggressive malignant salivary gland tumor characterized by a strong propensity for local invasion and an unfavorable prognosis. It carries a high risk of early local recurrence and both regional and distant metastasis.

## Introduction

Salivary duct carcinoma (SDC) is a high-grade malignancy originating from the ductal epithelium of the salivary glands, accounting for approximately 1-3% of all salivary gland tumors [1,2]. Histologically resembling invasive ductal carcinoma of the breast, it is characterized by intraductal tumor growth with comedo-type necrosis [3]. Due to its propensity for early recurrence and distant metastasis, the long-term survival rate is poor, with a five-year overall survival rate of 50-60% [4,5]. Despite significant advances in diagnostic and therapeutic technologies, the five-year survival rate remains unsatisfactory, particularly among patients with advanced-stage disease. In this study, clinical data from seven patients with SDC were retrospectively analyzed, and their clinical manifestations and pathological features were evaluated alongside a review of the relevant literature to enhance understanding of this malignancy.

## Materials and methods

Clinical data

Clinical data from patients diagnosed with SDC at the Department of Clinical Pathology of Northern Jiangsu People's Hospital, China, between 2016 and 2024 were retrospectively collected.

Inclusion and exclusion criteria

Inclusion Criteria

(1) All cases were diagnosed as SDC based on histopathological examination conducted by the Department of Clinical Pathology. (2) Complete clinical and pathological data were available for each case.

Exclusion Criteria

(1) Cases with an initial clinical suspicion or pathological diagnosis of SDC that were subsequently reclassified as other types of salivary gland tumors or non-salivary gland-derived tumors upon pathological review. (2) Cases with substantially incomplete clinical records or missing key pathological data that did not meet the requirements for analysis in this study. (3) Cases involving malignant tumors metastatic to the salivary gland from other primary sites.

Methods

The collected specimens were fixed in 10% formalin, dehydrated routinely, embedded in paraffin, and prepared into sections, which were then stained with hematoxylin and eosin (H&E) and observed under an optical microscope. Immunohistochemistry was used for immunosurface labeling, with primary antibodies including CKpan, CK8/18, CEA, EMA, AR, 34βE12, GATA3, P63, P40, calponin, S100, SMA, ER, PR, and HER2.

Primary and secondary outcomes

Primary Outcomes

(1) Basic characteristics of patients. (2) Location of tumor occurrence. (3) Clinical manifestations. (4) Pathological and immunophenotypic characteristics.

Secondary Outcomes

(1) General characteristics of tumors. (2) Microscopic examination. (3) Treatment method.

## Results

Clinical characteristics

We collected clinical data from seven cases of SDC, including six male patients and one female patient. At the time of diagnosis, the mean age was 58 ± 13.84 years (range: 36 to 73 years). Four tumors arose in the parotid gland (4/7), two in the submandibular gland (2/7), and one in the upper lip mucosa (1/7). The maximum tumor diameter was 8.4 cm. The chief complaint was most commonly the presence of a painless mass (6/7). Four lesions were located on the left side and three on the right side (Table 1).

**Table 1:** Clinical data of seven cases of salivary duct carcinoma

Case	Gender	Age, years	Region	Symptom	Maximum diameter of the tumor, cm	Surgical approach	Diagnostic tests	Prognosis
1	Male	48	Left submandibular gland	Painless mass gradually enlarges	2	Tumor resection	Immunohistochemistry and HE staining	Good prognosis, regular follow-up
2	Male	69	Right parotid gland	Painless mass gradually enlarges	2.7	Tumor resection and lymph node dissection	Immunohistochemistry and HE staining	Good prognosis, regular follow-up
3	Male	36	Right submandibular gland	Numbness of the right tongue	5	Tumor resection and lymph node dissection	Immunohistochemistry and HE staining	Good prognosis, regular follow-up
4	Male	45	Right parotid gland	Painless mass gradually enlarges	2.8	Tumor resection and lymph node dissection	Immunohistochemistry and HE staining	Good prognosis, regular follow-up
5	Male	62	Left parotid gland	Painless mass gradually enlarges	5	Tumor resection	Immunohistochemistry and HE staining	Good prognosis, regular follow-up
6	Female	73	Right upper lip	Painless mass gradually enlarges	1.5	Tumor resection	Immunohistochemistry and HE staining	Good prognosis, regular follow-up
7	Male	73	Left parotid gland	Painless mass gradually enlarges	8.4	Tumor resection and lymph node dissection	Immunohistochemistry and HE staining	Good prognosis, regular follow-up

Tumor gross

**Figure 1:**
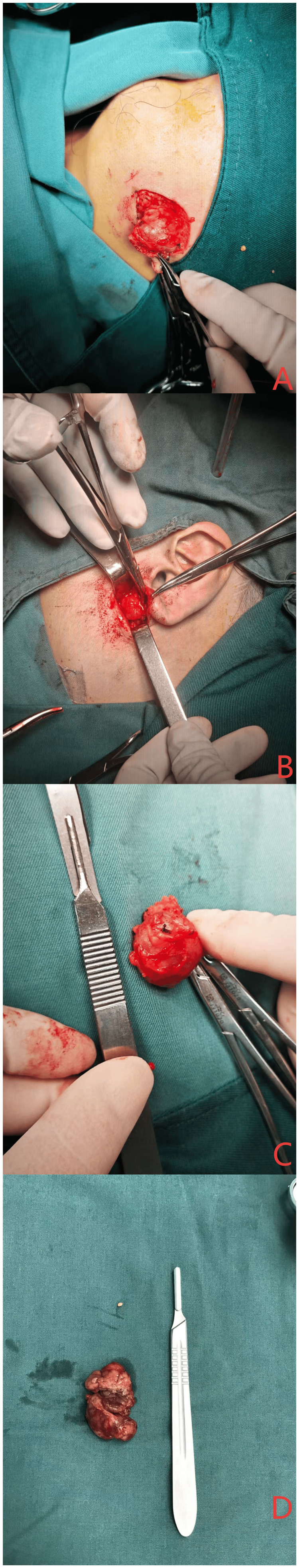
Intraoperative findings and resected specimens of salivary duct carcinoma From top to bottom: (A) Intraoperative exposure of the SDC lesion in the salivary gland region, showing a well-vascularized, solid red tumor mass with ill-defined borders and surrounding soft tissue involvement. (B) Meticulous surgical dissection of the tumor from adjacent normal tissues, with minimal intraoperative bleeding visualized in the operative field. (C) Gross appearance of the completely resected SDC specimen, presenting as a round, encapsulated mass with solid architecture, focal hemorrhage, and yellowish fatty tissue infiltration on the cut surface. (D) Another resected SDC specimen demonstrating an irregular, firm texture with extensive dark-red hemorrhagic and necrotic areas, consistent with the aggressive histopathological features of SDC.

The tumor was generally a solid nodular mass with unclear boundaries with surrounding tissues, with a diameter of 1.5-8.4 cm and no capsule. The cut surface is solid, with a hard texture and a grayish-white color (Figure 1).

Tumor microscopy examination

**Figure 2:**
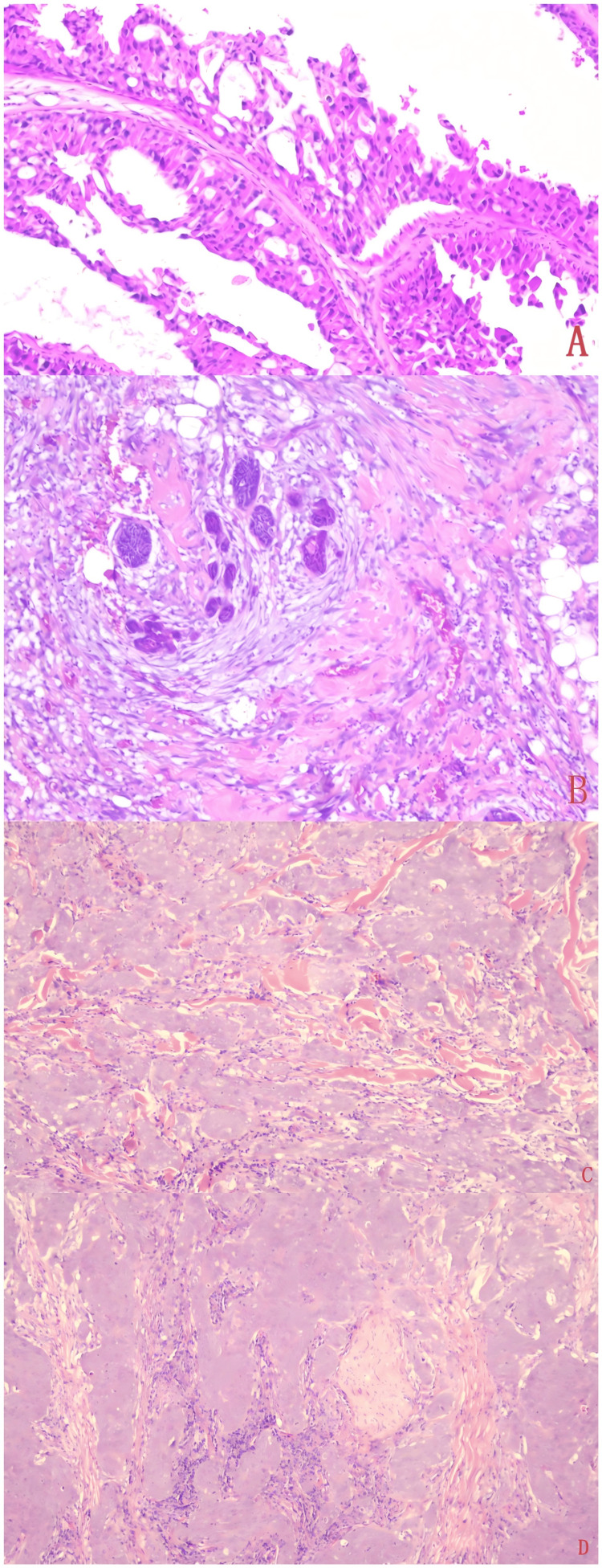
Macroscopic observation of salivary duct carcinoma tissue From top to bottom: (A) Salivary duct carcinoma tissue shows invasive growth. (B) Salivary duct carcinoma tissue shows necrosis. (C) Salivary duct carcinoma tissue invades striated muscle. (D) Salivary duct carcinoma tissue invades nerves.

The tumor tissue morphology of the seven patients was similar, resembling high-grade ductal carcinoma of the breast, with an invasive growth pattern (Figure 2A). Tumor cells were arranged in small glandular tubes, sieve-like structures, nests, and cords, and were mainly polygonal or cuboidal, with rich cytoplasm and eosinophilic staining. Significant cellular atypia was observed, with large nuclei and prominent nucleoli, and frequent mitotic figures. Partial necrosis could be seen within the lumen, accompanied by collagen deformation, and necrosis or calcification was also observed in the lesion area (Figure 2B). Partial invasion of nerves, surrounding tissues, and lymphatic vessels was noted (Figure 2C, Figure 2D).

Immune phenotype

**Figure 3:**
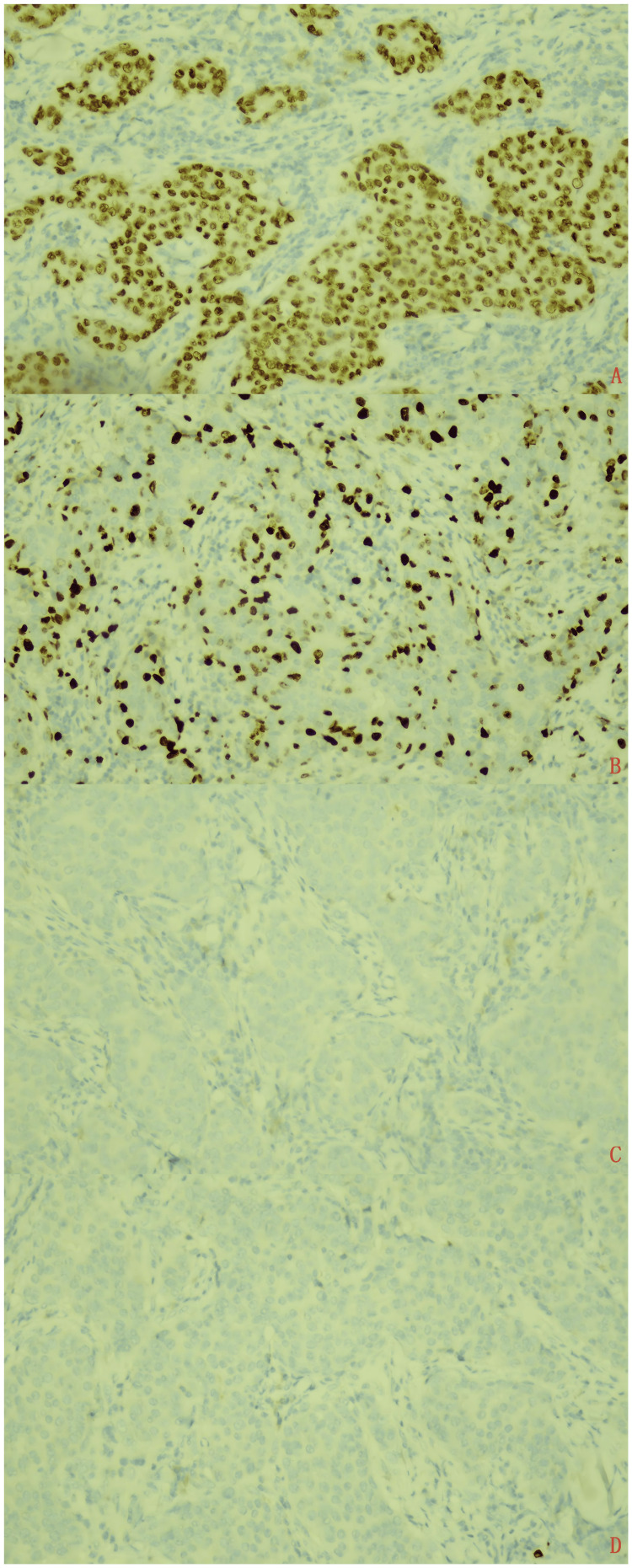
Immune phenotype of salivary duct carcinoma tissue From top to bottom: (A) The cells in salivary duct carcinoma tissue are positive for GATA3. (B) The cells in salivary duct carcinoma tissue are positive for Ki-67. (C) The cells in salivary duct carcinoma tissue are negative for P40. (D) The cells in salivary duct carcinoma tissue are negative for S100

All seven cases showed tumor cell positivity for CKpan, CK8/18, CEA, EMA, AR, 34βE12, and GATA3 (Figure 3A), with a Ki-67 (Figure 3B) proliferation index ranging from 15% to 70%. P63, P40 (Figure 3C), calponin, S100 (Figure 3D), SMA, and ER were all negative. One case (1/7) showed weak PR positivity, and two cases (2/7) demonstrated HER2 expression scored as 2+.

## Discussion

Salivary duct carcinoma is a highly malignant salivary gland epithelial tumor, first reported by Kleinsasser in 1968. The World Health Organization officially designated it as "salivary duct carcinoma" in the new histological classification of salivary gland tumors, and described it as an invasive adenocarcinoma resembling high-grade ductal carcinoma of the breast in 1990 [6,7].

Clinical features

The disease is more common in elderly men aged 60-70 years, with a male-to-female ratio of approximately 7:3. Most cases originate in the parotid gland, followed by the submandibular gland [8,9]. Clinically, the initial symptom is usually a rapidly enlarging, painless mass. It is prone to local recurrence, lymph node metastasis, and distant metastasis, with the lungs and bones being the most common sites. The prognosis for SDC is extremely poor. Relevant studies have shown that the five-year survival rate ranges from 30 to 64% [2,5,10-16]. In this study, the age range of the seven patients was 36-73 years, with an average age of 58 years. There were six males and one female. Four cases occurred in the parotid gland, two in the submandibular gland, and one in the upper lip mucosa.

Pathological features

On gross examination, the tumors typically appear grayish-white or grayish-red, soft or moderately firm, with ill-defined borders and possible invasion into surrounding tissues. Under the microscope, SDC demonstrates highly characteristic features, presenting as nests of cancer cells arranged in cribriform, papillary, or solid patterns, with central comedo-type necrosis. The tumor cells exhibit marked pleomorphism, varying in size and shape, and may be cuboidal, polygonal, or columnar. The nuclei are large with prominent nucleoli, frequent mitotic activity, irregular nuclear membranes, and abundant cytoplasm, often eosinophilic or granular. The tumor stroma often shows a pronounced desmoplastic reaction, with varying degrees of inflammatory cell infiltration, including lymphocytes and plasma cells. The tumor cells grow invasively, breaching the basement membrane and invading surrounding tissues such as nerves, blood vessels, and lymphatics. All seven cases in this study were consistent with these typical pathological features of SDC.

Immune phenotype

The immunophenotype of SDC has distinctive features and is closely related to diagnosis, differential diagnosis, and targeted therapy. SDC usually expresses CK, such as CKpan and CK8/18, as well as EMA, which confirms its origin from salivary duct epithelium. Some tumor cells of SDC can express CEA, which plays a supportive role in the diagnosis. 34βE12 is an immunohistochemical antibody combination of high molecular weight cytokeratins CK1, CK5, CK10, and CK14, which helps confirm the origin of ductal epithelium in SDC and indirectly indicates tumor differentiation and invasiveness based on the intensity of its expression [17]. The Ki-67 index is a key indicator for evaluating the proliferation activity of tumor cells, and its value is closely related to the malignancy, invasiveness, metastatic potential, and prognosis of the tumor [18].

A high Ki-67 index usually indicates increased tumor cell proliferative activity and is associated with tumor invasiveness, a higher recurrence rate, and poor prognosis. Related studies have shown that in the T1 and T2 groups of cancer patients, those with Ki-67 values of 15% or lower have a higher survival rate than those with Ki-67 values exceeding 15% [19]. Most patients with SDC express androgen receptor (AR), and the level of expression is typically high, which is closely related to prognosis. Related studies have shown that patients with AR-positive SDC have a relatively better prognosis, while those with AR-negative expression have a poorer prognosis [20].

GATA3 regulates the transcription of many genes in normal tissues and tumor cells and plays an important role in the diagnosis of various epithelial tumors. According to relevant studies, GATA3 expression is significant in SDC [21]. In SDC, markers such as P63, P40, calponin, S100, and SMA are usually negative or only weakly positive [22,23]. SDC generally does not express estrogen receptor (ER) or progesterone receptor (PR), although a few studies have suggested that low-level expression of ER and PR may occur [24,25]. SDC often overexpresses HER2, with the incidence of HER2-positive expression being approximately 40%, providing a potential therapeutic target and supporting consideration of HER2-targeted therapies [26].

Differential diagnosis

SDC can be diagnosed through typical pathological histology and immunophenotype, but its morphology and immunophenotype are similar to those of breast cancer and prostate cancer. Combined with clinical history and immunophenotype, if there is a history of primary breast or prostate cancer, metastatic cancer can be considered.

In addition, salivary duct carcinoma (SDC) needs to be distinguished from other high-grade salivary gland tumors: (1) High-grade mucoepidermoid carcinoma exhibits solid growth and can morphologically resemble SDC, with mucinous cells, epidermoid cells, and intermediate cells present. P63 and P40 are positive in mucoepidermoid carcinoma, while diffuse P63 or P40 positivity is extremely rare in SDC [6]. (2) Adenoid cystic carcinoma shows biphasic differentiation, with focal tubular or sieve-like growth. Cells are arranged in glandular or cystic structures, often accompanied by mucinous or clear changes. The nuclei are round or oval, nucleoli are inconspicuous, and mitotic figures are rare. S100, SOX10, CK7, and CAM5.2 are often diffusely positive, but AR is not expressed. In contrast, AR expression is often positive in SDC [23]. (3) Polymorphous adenocarcinoma is the most common benign salivary gland epithelial tumor, where epithelial and myoepithelial cells form sieve-like, trabecular, solid, tubular, and myoepithelial structures. It lacks significant cellular atypia and true ductal formation, whereas SDC usually shows obvious atypia and clear ductal differentiation [27]. (4) Intraductal carcinoma is a non-invasive tumor characterized by the proliferation of tumor cells within the duct. It can display sieve-like, papillary, solid, or cribriform patterns, with diverse tumor cell shapes including cuboidal, columnar, or flattened. These tumors usually express markers such as calponin and P63, confirming the integrity of the basement membrane [28]. In contrast, SDC tumor cells invade beyond the basement membrane, and P63 expression is usually negative.

Treatment and prognosis

Radical surgery is the primary treatment for salivary duct carcinoma. During surgery, the tumor should be completely excised whenever possible, and lymph node dissection should be performed to reduce the risk of recurrence. Postoperative comprehensive treatments, including radiotherapy, chemotherapy, targeted therapy, or immunotherapy, can improve patient survival. SDC is highly invasive and prone to recurrence and metastasis. Related studies have reported a recurrence rate of 43.33%, a metastasis rate of 46.67%, and overall survival rates at three and five years of 60.8% and 31.2%, respectively [29]. Bao et al. reported that CK5/6-positive patients are often at clinical stage III/IV and have a poor prognosis, necessitating close clinical monitoring [30].

Limitations

This study has several limitations. First, due to the low incidence of SDC, the number of cases included was small, resulting in a limited sample size. This may affect the representativeness and statistical reliability of the results, making it difficult to fully reflect the overall clinical and pathological characteristics of the disease. Second, the study was a single-center retrospective analysis, and all cases were sourced from the same medical institution, which may introduce selection bias and limit the generalizability of the conclusions. In addition, retrospective studies rely on existing medical records, and some clinical or follow-up information may be incomplete, with long-term follow-up data often lacking, making it impossible to systematically evaluate patient survival and recurrence over time. Although this study provides a preliminary description of immunophenotypic characteristics, it did not explore molecular biology or related mechanisms, and analysis of emerging topics such as targeted therapy remains limited. Future prospective multicenter studies with larger sample sizes, combined with molecular pathology techniques, are needed to further clarify the biological behavior of SDC and guide personalized treatment strategies.

In summary, this study aims to provide a scientific basis for the clinical diagnosis and pathological evaluation of SDC by conducting an in-depth analysis of its clinical features, pathological characteristics, immunophenotypes, differential diagnosis, treatment, and prognosis. The findings can offer valuable support for developing treatment strategies and improving patient outcomes.

## Conclusions

SDC is a rare but highly malignant salivary gland tumor with aggressive biological behavior. A retrospective analysis of seven cases in this study revealed that the tumor predominantly occurs in middle-aged and elderly males, most commonly in the parotid gland, and typically presents as a painless mass. Pathologically, it resembles high-grade ductal carcinoma of the breast, exhibiting significant cellular atypia and infiltrative growth. Immunohistochemical analysis shows a distinctive expression profile, including positivity for CKpan, CK8/18, AR, and GATA3, while being negative for P63, P40, S100, and other markers, with a high Ki-67 index. Some cases also demonstrate strong HER2 positivity, providing important diagnostic and differential diagnostic clues.

Although radical surgery combined with adjuvant therapy remains the primary treatment approach, SDC still exhibits a high recurrence rate and metastatic potential, resulting in a poor prognosis. This study highlights the importance of integrating clinical, morphological, and immunophenotypic analyses and identifies potential molecular markers for personalized targeted therapy, which may improve clinical management and patient outcomes. Future research with larger sample sizes is needed to further elucidate its molecular mechanisms and optimize treatment strategies.
